# Associations Between Body Roundness Index and Cardiovascular Outcomes: A China Kadoorie Biobank Prospective Cohort Study

**DOI:** 10.1016/j.jacasi.2025.05.014

**Published:** 2025-07-22

**Authors:** Chaoyue Zhao, Xushen Yang, Shiyu Zhu, Yufeng Wu, Gaoyu Yu, Hui Ni, Yini Shen, Meixiang Xiang, Yao Xie

**Affiliations:** a Department of Cardiology, The Second Affiliated Hospital, Zhejiang University School of Medicine, State Key Laboratory of Transvascular Implantation Devices, Heart Regeneration and Repair Key Laboratory of Zhejiang Province, Hangzhou, Zhejiang, China

**Keywords:** body roundness index, cardiovascular diseases, China Kadoorie Biobank, obesity, visceral fat

## Abstract

**Background:**

Obesity traditionally scaled by body mass index, and visceral fat are independent risk factors for cardiovascular diseases (CVDs). Assessing obesity’s impact on CVDs based solely on height or weight can be inaccurate. The body roundness index (BRI) estimates visceral fat distribution, but its association with CVDs in large-scale Chinese cohort remained unexplored.

**Objectives:**

This study sought to investigate the association between BRI and cardiovascular outcome in the general Chinese population.

**Methods:**

In a prospective cohort of 488,656 CKD (China Kadoorie Biobank) participants free of CVDs, multivariable Cox proportional hazard models were employed to investigate the association between BRI and a composite outcome of coronary heart disease (CHD), heart failure (HF), stroke, and cardiovascular death. The mediation effect of hypertension and diabetes in the BRI-CVD association was further explored.

**Results:**

During a median follow-up of 10.2 years (Q1-Q3: 9.2-11.3 years), 76,891 of 488,656 cases (15.7%) of composite outcome were documented. Compared to the lowest BRI quartile, participants in the highest quartile exhibited significantly increased hazards of composite outcomes (HR: 1.37; 95% CI: 1.34-1.40), CHD (HR: 1.52; 95% CI: 1.47-1.57), HF (HR: 1.24; 95% CI: 1.13-1.37), and stroke (HR: 1.41; 95% CI: 1.37-1.46). Restricted cubic spline analysis demonstrated J-shaped dose-response associations between BRI and composite outcome or CHD, whereas the BRI-HF and BRI-cardiovascular death associations were U-shaped. Both hypertension and diabetes mediated the BRI-CVD association, with a mediation proportion of 14.2% (95% CI: 13.2%-15.0%) and 1.7% (95% CI: 1.4%-2.0%), respectively.

**Conclusions:**

In the CKB cohort, BRI was positively significantly associated with cardiovascular outcome. Hypertension could be a potential mediator in the BRI-CVD association.

Obesity is an independent risk factor for cardiovascular diseases (CVDs),^1^^,^^2^ type 2 diabetes,^3^ and various chronic diseases. China has the most overweight and obese people in numbers globally, with 34.3% of adult residents, nearly 11.1% of children and adolescents aged 6-17 years, and even 6.8% of children under 6 years old being overweight or obese.^4^ Overweight and obese are strongly associated with noncommunicable diseases and premature mortality in Chinese populations.^4^ The growing burden of overweight and obese in China is now becoming a major social issue.

Body mass index (BMI), calculated as kg/m^2^, is commonly applied to scale the extent of obesity. Robust evidence supported a positive relationship between BMI and of all-cause mortality.^5^ In the US population, obesity was significantly associated with shorter longevity and increased risk of cardiovascular morbidity and mortality.^6^ Whereas BMI is well suited for population-level studies, solely applying it to describe obesity leads to inaccurate assessments of body fat, because BMI does not distinguish lean muscle from fat mass.^7^ Consequently, an individual with central obesity and a possibly normal BMI may still face a higher risk of mortality.^8^ Adipose distribution and muscle mass varied dramatically across genders and ages. Obesity in male subjects tends to manifest more as visceral fat accumulation, whereas in female subjects, it often appears as subcutaneous fat accumulation. Visceral adipose tissue accumulation results in elevated inflammatory levels and secretion of vasoconstrictor mediators, which lead to pathogenesis of CVDs, whereas expansion of subcutaneous adipose tissue seems less harmful.^9^ Therefore, evaluating the impact of obesity on CVDs based solely on height or weight is not accurate. Because there are limitations of BMI in representing the distribution of adipose tissue, an index reflecting abdominal and visceral adiposity is urgently needed.

The body roundness index (BRI) takes into account an individual's height and waist circumference (WC) to estimate visceral fat distribution.^10^ A recent study revealed a U-shaped association between BRI and all-cause mortality during 20-year period among US adults.^11^ In addition, BRI demonstrated superior over other obese indices, especially for BMI, in predicting the risk of cardiovascular disease. In the UROSAH (Urumqi Research on Sleep Apnea and Hypertension) study which was mainly composed of the patients from people’s hospital of Xinjiang Uygur autonomous region, BRI was significantly associated with increased risk of CVD in hypertensive patients with obstructive sleep apnea.^12^ By applying a population-based screening project in Southern China, BRI suggested much better performance in detecting cardiovascular multimorbidity compared to BMI.^13^ However, due to the respective limitations of specified population, sample size, cross-sectional design, and so on, the predictive value of BRI for CVD and mortality remains inconclusive in the general Chinese population.

In the present study, we aim to assess the association between BRI and cardiovascular outcomes in the general population, using large-scale prospective data from the CKB (China Kadoorie Biobank). Additionally, we examine the mediation effects of hypertension and diabetes in the BRI-CVD association in the Chinese population.

## Methods

### Study population

CKB is a nationwide cohort study with over half a million participants aged 30-79 years recruited from 10 geographically diverse areas (5 rural counties and 5 urban districts) in China.^14^ Specifically, the baseline survey was carried out between June 2004 and July 2008, where all participants completed interviewer-administered questionnaires, physical measurements, and blood sample collection. Participants are being followed for cause-specific mortality and morbidity, as well as for any hospital admission through linkages with registries and health insurance databases. Detailed information on CKB methodology and cohort profile is available elsewhere.^15^ The CKB protocol was approved by the ethics committees of the University of Oxford and the China National Center for Disease Control and Prevention. All participants provided written informed consent. This research has been conducted using the CKB resource (request no. DAR-2024-00423).

**Figure 1:**
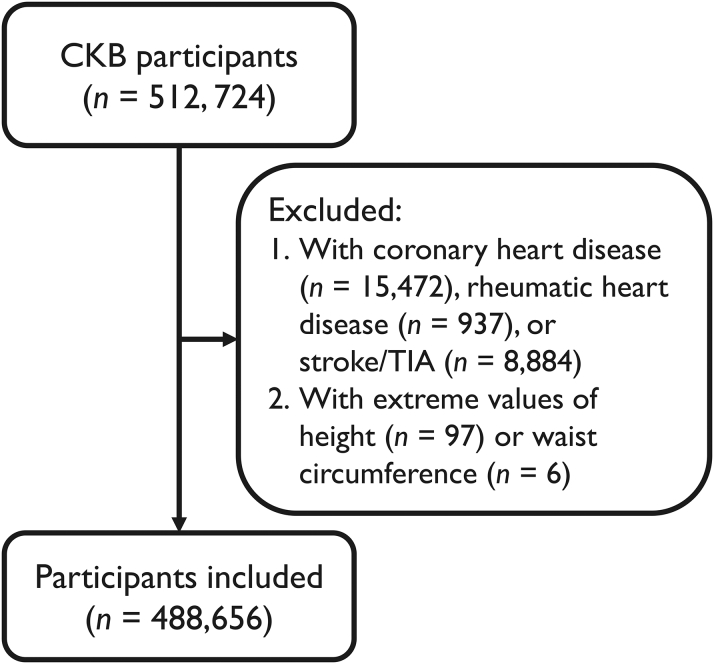
Study Flowchart The flowchart concisely presents the criteria for patient inclusion and exclusion. CKB = China Kadoorie Biobank; TIA = transient ischemic attack.

A total of 512,724 CKB participants were screened for eligibility. We exclude participants with self-reported coronary heart disease (CHD) (n = 15,472), stroke or transient ischemic attack (n = 8,884), or rheumatic heart disease (n = 937). Participants with extreme values in height (either <130 cm or >200 cm, n = 97) or WC (either <30 cm or >150 cm, n = 6) were also excluded in accordance with recommendations from the CKB team. Finally, 488,656 participants were included (Figure 1). Detailed definitions of exclusion criteria are shown in Supplemental Table 1.

### Assessment of BRI

Standing height and WC of study participants were measured during baseline assessment following standardized protocols. BRI was calculated with the following formula:^10^

$$BRI=364.2-365.5\times \sqrt{1-{\left(\frac{\frac{waist\;circumference}{2\pi }}{\frac{height}{2}}\right)}^{2}}$$

Due to the lack of established threshold, BRI was modeled as both continuous and categorical variables (in quartiles).

### Ascertainment of study outcomes

The primary outcome of the study is a composite outcome of incident CVDs including CHD, heart failure (HF), and stroke and cardiovascular death. Secondary outcomes consist of incident CHD, HF, and stroke, and cardiovascular mortality. Incident CVDs and cardiovascular death were identified through linkages to national health insurance claim database, disease and mortality registries, and supplemented with local residential records and annual active confirmation. The International Classification of Diseases, 10th Revision (ICD-10) codes used to define study outcomes were I20-I25 for CHD; I50 for HF; I60, I61, I63, and I64 for stroke; and I00-I25, I28-I88, and I95-I99 for cardiovascular death, respectively. Participants were censored until death, lost to follow-up, or the end of follow-up, whichever came first. Detailed definitions of study outcomes are shown in Supplemental Table 1.

### Covariates

Sociodemographic information (age, sex, marital status, educational level, and household income), medical history (diabetes and hypertension), and personal lifestyle (smoking, drinking, physical activity, diet, and sleep duration) were collected through electronical questionnaire during baseline assessment. Physical examinations were also performed with standardized protocols. Body fat percentages were measured with a bioelectrical impedance device (TANITA-TBF-300GS; Tanita Corp).^15^ Results of laboratory tests (liver and renal function, lipid profile, and inflammatory markers) were only publicly available in a small proportion of participants (n = 9,336). Detailed source and definitions for covariates are shown in Supplemental Table 1.

### Statistical analysis

Baseline characteristics stratified by BRI quartiles were summarized as mean ± SD or median (Q1-Q3) for continuous variables, and number (percentage) for categorical variables. Associations between BRI and other obesity-related indices and laboratory markers were explored using Pearson or Spearman correlation analysis where appropriate.

Kaplan-Meier curves of study outcomes stratified by BRI quartiles were depicted and compared with log-rank tests. Multivariable Cox proportional hazard models were employed to estimate the HRs and 95% CIs of BRI for study outcomes. A number of confounders were considered based on empirical evidence and previous published data,^16^^,^^17^ including age, sex, study region (urban/rural), marital status, educational level, household income, smoking, drinking, physical activity (metabolic equivalent), dietary pattern (as previously defined^16^), sleep duration, hypertension, and diabetes. We did not adjust for other obesity-related indices (such as BMI) to avoid collinearity with BRI. Proportional hazard assumptions were tested by Schoenfeld residuals, and no violations were detected. Based on sample size, restricted cubic spline analysis with 5 knots at 5th, 27.5th, 50th, 72.5^th^, and 95th percentiles were empirically used to investigate dose-response relationships between BRI and cardiovascular outcomes.^18^ Additionally, we performed subgroup analysis stratified by age, sex, study region, smoking, physical activity, dietary pattern, hypertension, and diabetes. Potential effect modification was tested by likelihood ratio test comparing the model with or without the cross-interaction term.

Mediation analyses were further employed to investigate mediation effect of hypertension and diabetes in the association between BRI and CVDs. The mediator models, linking BRI to hypertension or diabetes, were adjusted for age, sex, study region, marital status, educational level, household income, smoking, drinking, physical activity, dietary pattern, sleep duration, hypertension, and diabetes. The outcome model was constructed using the “survreg” function, assuming a Weibull distribution, with the same covariates as in the mediator model. The proportion mediated was estimated using the “mediate” function with 1,000 quasi-Bayesian simulations to derive CIs.

We also built univariable and multivariable Cox models to evaluate the strength of associations of BRI, BMI, WC, HC, and WHR with CVDs. Furthermore, we calculated the C-index of univariable Cox models to compare predictive capacity of these indices.

To validate the robustness of the study results, we performed the following sensitivity analysis: 1) excluding participants with CVD events within 2 years since follow-up to avoid reverse causality; 2) performing competing risk model analysis of Fine and Gray to account for the competing risk of noncardiovascular death; 3) additionally adjusting for the use of antihypertensive, lipid-lowering, and antidiabetic medications; 4) additionally adjusting for low-density lipoprotein-cholesterol in the subgroup with available laboratory test result.

All statistical analyses were performed in R (version 4.4.1, R Foundation), and the “mediation” package was used in the mediation analysis. A 2-tailed *P* < 0.05 was considered statistically significant.

## Results

Among the 488,656 included participants, BRI was normally distributed with a range of −0.34 to 18.71, a mean of 3.52 ± 1.16 (Supplemental Figure 1). Baseline characteristics of study participants are summarized in Table 1 and Supplemental Table 2. Participants in higher BRI quartiles were older, more likely to be female subjects, have a higher prevalence of diabetes and hypertension, but were less likely to be a current smoker (all *P* < 0.001). Correlation analysis illustrated positive associations among BRI and body fat percentage, low-density lipoprotein-cholesterol, and hypersensitive C-reactive protein levels (Supplemental Figure 2).

**Table 1:** Baseline Characteristics of Study Participants Stratified by BRI Quartiles

	Overall (N = 488,656)	Q1 <2.66 (n = 122,164)	Q2 2.66-3.39 (n = 122,168)	Q3 3.39-4.22 (n = 122,161)	Q4 >4.22 (n = 122,163)	*P* Value
Sociodemographic variables						
Age, y	50.98 (42.63-58.95)	48.70 (40.69-57.36)	49.39 (41.74-57.14)	51.09 (43.19-58.66)	54.18 (46.53-62.00)	<0.001
Female sex	288,710 (59.1)	62,820 (51.4)	70,544 (57.7)	72,312 (59.2)	83,034 (68.0)	<0.001
Study region						<0.001
Rural	277,631 (56.8)	77,675 (63.6)	71,868 (58.8)	65,368 (53.5)	62,720 (51.3)	
Urban	211,025 (43.2)	44,489 (36.4)	50,300 (41.2)	56,793 (46.5)	59,443 (48.7)	
Marital status						<0.001
Married	444,099 (90.9)	111,330 (91.1)	112,058 (91.7)	111,706 (91.4)	109,005 (89.2)	
Widowed	33,288 (6.8)	6,955 (5.7)	7,015 (5.7)	7,982 (6.5)	11,336 (9.3)	
Separated/divorced	7,627 (1.6)	2,350 (1.9)	2,162 (1.8)	1,784 (1.5)	1,331 (1.1)	
Never married	3,642 (0.7)	1,529 (1.3)	933 (0.8)	689 (0.6)	491 (0.4)	
Education level						<0.001
No formal school	91,178 (18.7)	21,313 (17.4)	20,561 (16.8)	21,579 (17.7)	27,725 (22.7)	
Primary school	157,193 (32.2)	39,696 (32.5)	38,370 (31.4)	37,530 (30.7)	41,597 (34.1)	
Middle school	138,866 (28.4)	35,383 (29.0)	36,189 (29.6)	35,860 (29.4)	31,434 (25.7)	
High school	73,663 (15.1)	18,568 (15.2)	19,785 (16.2)	19,527 (16.0)	15,783 (12.9)	
College/university	27,756 (5.7)	7,204 (5.9)	7,263 (5.9)	7,665 (6.3)	5,624 (4.6)	
Annual household income, yuan						<0.001
<5,000	47,721 (9.8)	12,441 (10.2)	11,667 (9.5)	11,005 (9.0)	12,608 (10.3)	
5,000-9,999	90,723 (18.6)	23,865 (19.5)	23,206 (19.0)	21,439 (17.5)	22,213 (18.2)	
10,000-19,999	141,072 (28.9)	34,234 (28.0)	34,632 (28.3)	34,998 (28.6)	37,208 (30.5)	
20,000-34,999	120,749 (24.7)	30,651 (25.1)	30,611 (25.1)	30,717 (25.1)	28,770 (23.6)	
≥35,000	88,391 (18.1)	20,973 (17.2)	22,052 (18.1)	24,002 (19.6)	21,364 (17.5)	
Lifestyle and medical history						
Smoking status						<0.001
Never	302,936 (62.0)	67,028 (54.9)	74,643 (61.1)	76,865 (62.9)	84,400 (69.1)	
Occasional	27,965 (5.7)	6,572 (5.4)	7,168 (5.9)	7,635 (6.2)	6,590 (5.4)	
Past	27,221 (5.6)	5,692 (4.7)	6,182 (5.1)	7,387 (6.0)	7,960 (6.5)	
Current	130,534 (26.7)	42,872 (35.1)	34,175 (28.0)	30,274 (24.8)	23,213 (19.0)	
Drinking status						<0.001
Never	223,260 (45.7)	54,015 (44.2)	54,565 (44.7)	54,595 (44.7)	60,085 (49.2)	
Past	7,911 (1.6)	2,335 (1.9)	1,891 (1.5)	1,827 (1.5)	1,858 (1.5)	
Current	257,485 (52.7)	65,814 (53.9)	65,712 (53.8)	65,739 (53.8)	60,220 (49.3)	
Physical activity (metabolic equivalent)	18.04 (10.71-30.53)	21.29 (12.32-33.75)	19.64 (11.29-32.01)	17.48 (10.63-29.85)	14.50 (8.90-25.80)	<0.001
Healthy diet	8,509 (1.7)	1,802 (1.5)	2,018 (1.7)	2,177 (1.8)	2,512 (2.1)	<0.001
Sleep duration, h						<0.001
≤6	110,438 (22.6)	26,295 (21.5)	26,532 (21.7)	27,617 (22.6)	29,994 (24.6)	
7-8	300,113 (61.4)	75,403 (61.7)	76,142 (62.3)	75,579 (61.9)	72,989 (59.7)	
≥9	78,105 (16.0)	20,466 (16.8)	19,494 (16.0)	18,965 (15.5)	19,180 (15.7)	
Diabetes	26,363 (5.4)	2,597 (2.1)	4,382 (3.6)	7,152 (5.9)	12,232 (10.0)	<0.001
Hypertension	159,222 (32.6)	24,986 (20.5)	32,157 (26.3)	42,943 (35.2)	59,136 (48.4)	<0.001
Physical examination						
SBP, mm Hg	130.61 ± 21.05	124.20 ± 19.46	127.79 ± 19.73	132.14 ± 20.56	138.30 ± 21.72	<0.001
DBP, mm Hg	77.66 ± 11.10	74.62 ± 10.53	76.49 ± 10.69	78.74 ± 10.95	80.77 ± 11.23	<0.001
Height, cm	158.70 ± 8.26	160.29 ± 7.88	159.02 ± 8.07	158.67 ± 8.36	156.80 ± 8.34	<0.001
Weight, kg	59.62 ± 10.70	52.13 ± 7.49	57.14 ± 8.29	61.79 ± 9.35	67.43 ± 10.84	<0.001
Waist circumference, cm	80.05 ± 9.67	69.29 ± 4.68	76.62 ± 4.28	82.78 ± 4.73	91.51 ± 6.72	<0.001
Hip circumference, cm	90.78 ± 6.80	85.44 ± 4.92	89.01 ± 4.99	92.09 ± 5.25	96.60 ± 6.40	<0.001
Obesity-related indices						
BMI, kg/m^2^	23.60 ± 3.36	20.21 ± 1.83	22.49 ± 1.79	24.41 ± 1.98	27.29 ± 2.76	<0.001
Waist-hip ratio	0.88 ± 0.07	0.81 ± 0.05	0.86 ± 0.05	0.90 ± 0.05	0.95 ± 0.06	<0.001
Waist-height ratio	0.50 ± 0.06	0.43 ± 0.02	0.48 ± 0.01	0.52 ± 0.01	0.58 ± 0.04	<0.001
Body fat percentage	27.86 ± 8.35	20.67 ± 5.89	25.85 ± 6.07	29.62 ± 6.28	35.31 ± 7.35	<0.001

Values are mean ± SD, median (Q1-Q3), or n (%).

BMI = body mass index; BRI = body roundness index; DBP = diastolic blood pressure; SBP = systolic blood pressure.

**Figure 2:**
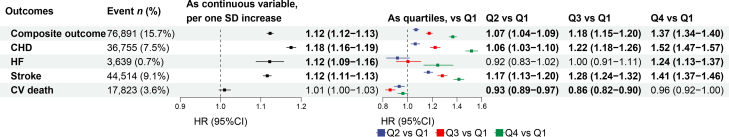
Associations Among BRI and CV Outcomes The HRs (95% CIs) were adjusted for age, sex, study region, marital status, educational level, household income, smoking, drinking, physical activity, dietary pattern, sleep duration, hypertension, and diabetes. BRI = body roundness index; CHD = coronary heart disease; CV = cardiovascular; HF = heart failure.

During a median follow-up of 10.2 years (Q1-Q3: 9.2-11.3 years; 4.9 million person-years), a total of 76,891 of 488,656 cases (15.7%) of composite outcome, 36,755 of 488,656 cases (7.5%) of CHD, 3,639 of 488,656 cases (0.7%) of HF, 44,514/ of ,656 cases (9.1%) of stroke, and 17,823 of 488,656 cases (3.6%) of cardiovascular death were documented (Supplemental Figure 3). In the multivariable Cox proportional hazard models (Figure 2), BRI was positively associated with the risk of composite outcome, as well as incident CHD, HF, and stroke (all *P* < 0.001) but not cardiovascular death. Compared to the lowest BRI quartile, participants in the highest quartile exhibited significantly increased hazards of composite outcomes (HR: 1.37; 95% CI: 1.34-1.40), CHD (HR: 1.52; 95% CI: 1.47-1.57), HF (HR: 1.24; 95% CI: 1.13-1.37), and stroke (HR: 1.41; 95% CI: 1.37-1.46). On the contrary, participants in the middle 2 quartiles of BRI were observed to have lower risks of cardiovascular death (HR: 0.93; 95% CI: 0.89-0.97; and HR: 0.86; 95% CI: 0.82−0.90, respectively).

**Figure 3:**
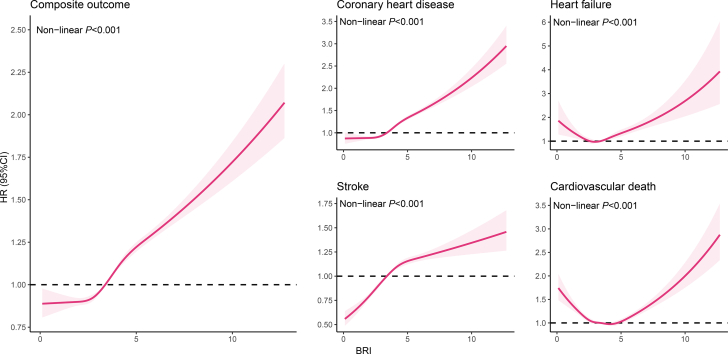
Restricted Cubic Splines of BRI for CV Outcomes Adjusted for the same covariates as in Figure 2. BRI = body roundness index; other abbreviation as in Figure 2.

Restricted cubic spline analysis (Figure 3) further illustrated a J-shaped dose-response association between BRI and composite outcome as well as CHD (nonlinear *P* < 0.001). Meanwhile, the BRI-HF and BRI-cardiovascular death association were U-shaped (nonlinear *P* < 0.001).

In subgroup analysis, the BRI-CVD association remained significant among different subgroups (Supplemental Figure 4). Of note, potential effect modification of age on the BRI-CVD association was found, where the young were more susceptible to CVDs when exposed to higher levels of BRI (all *P* for effect modification < 0.001).

When compared to conventional anthropometric measures such as BMI, WHR, WC, and HC, BRI showed a stronger strength of association and modest improvement in predictive capacity of CVDs in univariable analysis (Supplemental Tables 3 and 4).

The mediation effect of hypertension and diabetes in the BRI-CVD association is presented in Table 2. Both hypertension and diabetes potentially mediated the BRI-CVD association, with a mediation proportion of 14.2% (95% CI: 13.2%-15.0%) and 1.7% (95% CI: 1.4%-2.0%), respectively.

**Table 2:** Results of the Mediation Analysis

Mediator	Hypertension	Diabetes
Total effect	−1,785 (−1,900 to −1,668), *P* < 0.001	−1,490 (−1,604 to −1,369), *P* < 0.001
Direct effect	−1,532 (−1,645 to −1,421), *P* < 0.001	−1,465 (−1,579 to −1,345), *P* < 0.001
Indirect effect	−253 (−266 to −240), *P* < 0.001	−25 (−28 to −22), *P* < 0.001
Proportion mediated, %	14.2 (13.2-15.0)	1.7 (1.4-2.0)

Values are median (Q1-Q3).

In the sensitivity analysis, excluding participants with CVD events within 2 years since follow-up, using the competing risk model or additionally adjusting medication use did not significantly alter the study results (Supplemental Table 5), and additionally adjusting low-density lipoprotein-cholesterol did not alter the main conclusions (Supplemental Table 6).

## Discussion

In a large-scale nationwide prospective cohort of the general Chinese population, we uncovered a significant association between BRI and cardiovascular outcome. Interestingly, heterogenous dose-response relationship between BRI and CHD, HF, and cardiovascular death was found. BRI was superior to BMI, WHR, WC, and HC in predicting cardiovascular outcomes. Furthermore, mediation analysis demonstrated a significantly higher mediation proportion of hypertension than diabetes in the BRI-CVD association (Central Illustration). Collectively, our findings provided the evidence of BRI in the risk stratification and precise prediction of CVDs in the Chinese population.

**Central Illustration:**
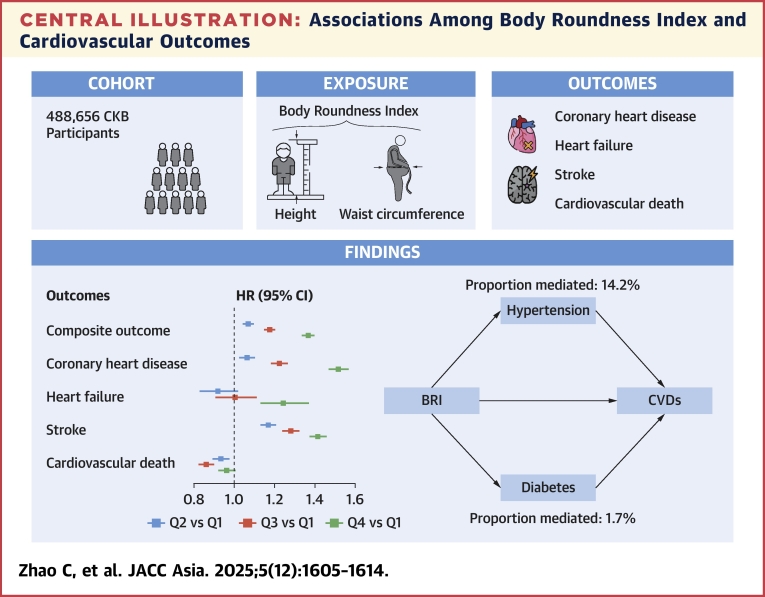
Associations Among Body Roundness Index and Cardiovascular Outcomes This figure illustrates the association between body roundness index (BRI) and cardiovascular outcomes in 488,656 CKB (China Kadoorie Biobank) participants free of cardiovascular diseases (CVDs). BRI was associated with the risk of composite outcome, as well as incident coronary heart disease, heart failure, stroke and cardiovascular death. Both hypertension and diabetes mediated the BRI-CVD association.

Visceral obesity, or exceeded storage of visceral adipose tissue, negatively contributed to cardiovascular events^19^^,^^20^ and all-cause mortality.^21^ The underlying mechanism might be partially attributed to the difference of stromal vascular fraction between subcutaneous and visceral adipose tissue.^22^ In addition, visceral fat accumulation was related to insulin resistance, glucose intolerance, elevated levels of C-reactive protein and tumor necrosis factor-α, all of which may further exacerbate CVDs.^23^ Therefore, a simple and effective anthropometric measure, which can represent visceral fat distribution to some extent, is urgently needed. BRI is a superior indicator for abdominal adiposity, especially when compared to BMI and other conventional indices. In addition, BRI has demonstrated its advantages in predicting various chronic obesity-related diseases.^13^^,^^24^^,^^25^ Our study also highlighted the advantage of BRI in predicting CVDs compared to BMI, WHR, WC, and HC. These findings suggest that BRI holds promise as an emerging tool for risk stratification and precise prediction of cardiovascular diseases.

Few studies investigated the association between BRI and CVDs as well as cardiovascular or all-cause mortality in the general Chinese population. In 9,935 participants from the CHARLS (China Health and Retirement Longitudinal Study), the BRI trajectory was associated with self-report heart diseases and stroke.^26^ In a retrospective cohort study with 71,166 participants from Henan Province, China, Ding et al^27^ identified a U-shaped relationship between BRI, and all-cause and cardiovascular mortality in patients with hypertension. Not only in Chinese population, by extracting the National Health and Nutrition Examination Survey (NHANES) data, a U-shaped association was identified between BRI and all-cause mortality in US adults.^11^ In a longitudinal population-based cohort study of 59,278 Chinese participants with repeated measurements of BRI from 2006 to 2012, the trajectories of BRI were significantly linked to the risk of CVD in a dose-dependent manner, particularly in younger adults.^28^ In our study, we discovered a U-shaped association between BRI and cardiovascular death as well as HF in a large-scale prospective cohort of Chinese general population, which strengthened the evidence for such dose-response relationship. On the contrary, the Kailuan cohort discovered a linear dose-response relationship between BRI and HF.^29^ The discrepancy might be explained by the different sex composition where over 80% of the Kailuan cohort participants are male subjects.^29^ To be noted, a J-shaped association between BRI, and composite outcome as well as CHD was identified for the first time, indicating the difference in predictive value of BRI in respective atherosclerotic cardiovascular diseases. The discrepancy in the shape of dose-response relationship in different BRI-CVD associations might reflect differences in underlying mechanisms: HF risk could be influenced by competing pathways (eg, malnutrition or sarcopenia at low BRI vs metabolic dysfunction at high BRI), whereas CHD may be more directly driven by obesity-related metabolic dysfunction.^30^, ^31^, ^32^, ^33^ The finding also suggested the potential cardiovascular benefit of reducing visceral adiposity, particularly for those with severe abdominal obesity. Given the heightened cardiovascular risks observed in individuals with the lowest BRI, greater attention should be directed toward this population, with further research needed to elucidate the underlying mechanisms and inform targeted interventions.

In subgroup analysis, we found that the BRI-CVD association was more evident among younger participants. It can be partly attributed to the fact that younger adults with higher levels of body fat may have an accelerated progression of the atherosclerotic process, which would consequently increase the risk of CVD.^34^ In addition, aging itself introduced competing risks (eg, noncardiovascular mortality, frailty) that may dilute the observed effect of BRI in older adults.^35^

In our mediation analysis, we noticed that both hypertension and diabetes mediated the BRI-CVD association, although the mediation effect is not that large for diabetes mellitus. This result is not surprising, and previous studies have illustrated the potential relationship between BRI and hypertension as well as diabetes. In a systematic review and meta-analysis of observational studies, BRI displayed discriminatory power for predicting hypertension.^36^ Liu et al^24^ recruited 6,990 hypertensive adults without diabetes in Dongguan City, China. Throughout a median follow-up period of 3 years, BRI stood out as the most superior predictor and an independent factor for the development of diabetes within the hypertensive population, when compared to BMI, WC, HC, WHR, waist-to-height ratio, and other novel indices (a body shape index, abdominal volume index, body adiposity index, conicity index, weight-adjusted-waist-index, and waist-hip-height ratio). Our data further solidified the previous conclusions and provided mechanistic insights into the association between visceral adiposity and CVDs.

### Study strengths and limitations

To our best knowledge, the CKB is the largest community-based prospective cohort study with a long-term follow-up in the Chinese general population to investigate the association between BRI and various atherosclerotic cardiovascular diseases. The representativeness of the CKB cohort further strengthened the evidence.^26^ There are still several limitations to be taken into account when interpreting the study results. First, BRI was only measured at baseline. We are unable to explore the association between longitudinal trajectories in BRI and CVDs. Second, the observational nature of the study could not conclude a causal relationship. The reliance on a single baseline measurement of BRI and the lack of repeated assessments for hypertension/diabetes during follow-up also preclude definitive conclusions about temporal mediation effect. Third, other confounders, which were not included either in this study or in CKB, could affect our result. In addition, we were not able to exclude participants with HF at baseline, which introduced bias when assessing the BRI-HF association. Finally, the CKB cohort mainly consisted of middle-aged and older Chinese population, which cannot be generalized to other ethnic populations.

## Conclusions

In the CKB cohort, BRI was positively significantly associated with cardiovascular outcome. Both hypertension and diabetes could be potential mediators in the BRI-CVD association. Our finding supported the role of BRI in the risk stratification and targeted prevention of CVDs within the Chinese population.

## Funding Support and Author Disclosures

This research is supported by The Natural Science Foundation of Zhejiang province, China (grants LZ23H070001 to Y.X.), and Provincial and Ministry Joint Major Projects of National Health Commission of China (grant WKJ-ZJ-2405 to M.X.). The authors have reported that they have no relationships relevant to the contents of this paper to disclose.

## References

[1] Zhao D. (2021). Epidemiological features of cardiovascular disease in Asia. JACC Asia.

[2] Wu F., Juonala M., Sabin M.A. (2020). Association of body mass index in youth with adult cardiometabolic risk. J Am Heart Assoc.

[3] Karin A., Jon E., Martin A. (2022). Body mass index in adolescence, risk of type 2 diabetes and associated complications: a nationwide cohort study of men. EClinicalMedicine.

[4] Pan X.F., Wang L., Pan A. (2021). Epidemiology and determinants of obesity in China. Lancet Diabetes Endocrinol.

[5] Flegal K.M., Kit B.K., Orpana H., Graubard B.I. (2013). Association of all-cause mortality with overweight and obesity using standard body mass index categories: a systematic review and meta-analysis. JAMA.

[6] Khan S.S., Ning H., Wilkins J.T. (2018). Association of body mass index with lifetime risk of cardiovascular disease and compression of morbidity. JAMA Cardiol.

[7] Cornier M.A., Després J.P., Davis N. (2011). Assessing adiposity: a scientific statement from the American Heart Association. Circulation.

[8] Coutinho T., Goel K., Corrêa de Sá D. (2011). Central obesity and survival in subjects with coronary artery disease: a systematic review of the literature and collaborative analysis with individual subject data. J Am Coll Cardiol.

[9] Koenen M., Hill M.A., Cohen P., Sowers J.R. (2021). Obesity, adipose tissue and vascular dysfunction. Circ Res.

[10] Thomas D.M., Bredlau C., Bosy-Westphal A. (2013). Relationships between body roundness with body fat and visceral adipose tissue emerging from a new geometrical model. Obesity (Silver Spring).

[11] Zhang X., Ma N., Lin Q. (2024). Body roundness index and all-cause mortality among US adults. JAMA Netw Open.

[12] Cai X., Song S., Hu J. (2023). Body roundness index improves the predictive value of cardiovascular disease risk in hypertensive patients with obstructive sleep apnea: a cohort study. Clin Exp Hypertens.

[13] Qin X., Chen C., Wang J. (2023). Association of adiposity indices with cardiometabolic multimorbidity among 101,973 Chinese adults: a cross-sectional study. BMC Cardiovasc Disord.

[14] Chen Z., Chen J., Collins R. (2011). China Kadoorie Biobank of 0.5 million people: survey methods, baseline characteristics and long-term follow-up. Int J Epidemiol.

[15] Chen Z., Lee L., Chen J. (2005). Cohort profile: the Kadoorie Study of Chronic Disease in China (KSCDC). Int J Epidemiol.

[16] Han Y., Hu Y., Yu C. (2021). Lifestyle, cardiometabolic disease, and multimorbidity in a prospective Chinese study. Eur Heart J.

[17] Lloyd-Jones D.M., Allen N.B., Anderson C.A.M. (2022). Life's essential 8: updating and enhancing the American Heart Association's construct of cardiovascular health: a presidential advisory from the American Heart Association. Circulation.

[18] Harrell F.E. (2016). Survival Analysis.

[19] Antonio-Villa N.E., Juárez-Rojas J.G., Posadas-Sánchez R., Reyes-Barrera J., Medina-Urrutia A. (2023). Visceral adipose tissue is an independent predictor and mediator of the progression of coronary calcification: a prospective sub-analysis of the GEA study. Cardiovasc Diabetol.

[20] Ruiz-Castell M., Samouda H., Bocquet V., Fagherazzi G., Stranges S., Huiart L. (2021). Estimated visceral adiposity is associated with risk of cardiometabolic conditions in a population based study. Sci Rep.

[21] Saad R.K., Ghezzawi M., Horanieh R. (2022). Abdominal visceral adipose tissue and all-cause mortality: a systematic review. Front Endocrinol (Lausanne).

[22] Xie Y., Ji Y., Lu Y. (2022). Distinct characteristics between perivascular and subcutaneous adipose-derived stem cells. Diabetes.

[23] Hocking S., Samocha-Bonet D., Milner K.L., Greenfield J.R., Chisholm D.J. (2013). Adiposity and insulin resistance in humans: the role of the different tissue and cellular lipid depots. Endocr Rev.

[24] Liu Y., Liu X., Guan H. (2021). Body roundness index is a superior obesity index in predicting diabetes risk among hypertensive patients: a prospective cohort study in China. Front Cardiovasc Med.

[25] Yang T., Zhao B., Pei D. (2021). Evaluation of the association between obesity markers and type 2 diabetes: a cohort study based on a physical examination population. J Diabetes Res.

[26] Yang M., Liu J., Shen Q. (2024). Body roundness index trajectories and the incidence of cardiovascular disease: evidence from the China Health and Retirement Longitudinal Study. J Am Heart Assoc.

[27] Ding J., Chen X., Shi Z., Bai K., Shi S. (2023). Association of body roundness index and its trajectories with all-cause and cardiovascular mortality among a Chinese middle-aged and older population: a retrospective cohort study. Front Public Health.

[28] Wu M., Yu X., Xu L., Wu S., Tian Y. (2022). Associations of longitudinal trajectories in body roundness index with mortality and cardiovascular outcomes: a cohort study. Am J Clin Nutr.

[29] Wang J., Wu M., Wu S., Tian Y. (2022). Relationship between body roundness index and the risk of heart failure in Chinese adults: the Kailuan cohort study. ESC Heart Fail.

[30] Ouchi N., Parker J.L., Lugus J.J., Walsh K. (2011). Adipokines in inflammation and metabolic disease. Nat Rev Immunol.

[31] He X., Zhu J., Liang W. (2025). Association of body roundness index with cardiovascular disease in patients with cardiometabolic syndrome: a cross-sectional study based on NHANES 2009-2018. Front Endocrinol (Lausanne).

[32] Lopes H.F., Corrêa-Giannella M.L., Consolim-Colombo F.M., Egan B.M. (2016). Visceral adiposity syndrome. Diabetol Metab Syndr.

[33] Powell-Wiley T.M., Poirier P., Burke L.E. (2021). Obesity and cardiovascular disease: a scientific statement from the American Heart Association. Circulation.

[34] Chung S.T., Krenek A., Magge S.N. (2023). Childhood obesity and cardiovascular disease risk. Curr Atheroscler Rep.

[35] Tian F., Chen L., Qian Z.M. (2023). Ranking age-specific modifiable risk factors for cardiovascular disease and mortality: evidence from a population-based longitudinal study. EClinicalMedicine.

[36] Calderón-García J.F., Roncero-Martín R., Rico-Martín S. (2021). Effectiveness of body roundness index (BRI) and a body shape index (ABSI) in predicting hypertension: a systematic review and meta-analysis of observational studies. Int J Environ Res Public Health.

