# Supplementary material for: Associations Between Body Roundness Index and Cardiovascular Outcomes: A China Kadoorie Biobank Prospective Cohort Study
Source: JACC Asia. 2025 Jul 22;5(12):1605–14. doi: 10.1016/j.jacasi.2025.05.014 (PMC12794001; doi:10.1016/j.jacasi.2025.05.014)
Supplement: Supplemental Figures 1-4 and Supplemental Tables 1-6 [file mmc1.docx]

**Associations Between Body Roundness Index and Cardiovascular Outcomes: A China Kadoorie Biobank Prospective Cohort Study**

Chaoyue Zhao^a#^; Xushen Yang^a#^; Shiyu Zhu, MD^a^; Yufeng Wu^a^; Gaoyu Yu^a^; Hui Ni, MD^a^; Yini Shen^a^; Meixiang Xiang MD, PhD^a^*, Yao Xie MD, PhD, MPH^a^*

^a^ Department of Cardiology, The Second Affiliated Hospital, Zhejiang University School of Medicine, State Key Laboratory of Transvascular Implantation Devices, Heart Regeneration and Repair Key Laboratory of Zhejiang Province, 88 Jiefang Road, Hangzhou, Zhejiang, 310009, China

*Correspondence:

Yao Xie, M.D., Ph.D., M.PH., E-mail: [xieyao@zju.edu.cn](mailto:xieyao@zju.edu.cn), Phone: +86-17857323088, Address: 88 Jiefang Road, Hangzhou, Zhejiang, 310009, China

Meixiang Xiang, M.D., Ph.D., E-mail: xiangmx@zju.edu.cn, Phone: +86-13588035801, Address: 88 Jiefang Road, Hangzhou, Zhejiang, 310009, China

#These authors contributed equally and shared the first authorship.

**Supplemental Materials**

**Supplemental Table 1.** Detailed definitions and source of study variables. 3

**Supplemental Table 2.** Baseline laboratory test results of study participants stratified by BRI quartiles (*n* = 9,336). 7

**Supplemental Table 3.** Relationship of obesity-related indices and cardiovascular outcomes. 9

**Supplemental Table 4.** C-index (95%CI) for Cox models of obesity-related indices and cardiovascular outcomes. 12

**Supplemental Table 5.** Results of the sensitivity analyses. 13

**Supplemental Table 6.** Cox Model Analysis Adjusted for LDL-C (*n* = 9336). 15

**Supplemental Figure 1.** Histogram of BRI in the CKB cohort. 16

**Supplemental Figure 2.** Results of the correlation analysis. 17

**Supplemental Figure 3.** Kaplan-Meier curves of BRI quartiles for cardiovascular outcomes. 18

**Supplemental Figure 4.** Results of the stratified analysis. 20

**Supplemental Table 1.** Detailed definitions and source of study variables.

| **Variable** | **Definition / Description** | **Source** |
| --- | --- | --- |
| **Exclusion criteria** | | |
| Previous coronary heart disease | Self-reported | chd_diag |
| Previous stroke or TIA | Self-reported | stroke_or_tia_diag |
| Previous rheumatic heart disease | Self-reported | rheum_heart_dis_diag |
| **Exposure** | | |
| Body roundness index | $BRI=364.2-365.5\times\sqrt{1-\left( \frac{\frac{waist}{2\pi}}{\frac{height}{2}} \right)^{2}}$ | Waist: waist_mm  Height: standing_height_mm |
| **Outcomes:** identified through linkages to disease and mortality registries and national health insurance claim database, supplemented with local residential records and annual active confirmation. | | |
| Coronary heart disease | ICD-10: I20-I25 (CKB0003) | ep_CKB0003_combined_ep  ep_CKB0003_combined_datedeveloped |
| Heart failure | ICD-10: I50 (CKB0080) | ep_CKB0080_combined_ep  ep_CKB0080_combined_datedeveloped |
| Stroke | ICD-10: I60, I61, I63, I64 (CKB0070) | ep_CKB0070_combined_ep  ep_CKB0070_combined_datedeveloped |
| Cardiovascular death | ICD-10: I00 to I25, I28 to I88, and I95 to I99 (CKB0104) | ep_CKB0104_combined_ep  ep_CKB0104_combined_datedeveloped |
| Composite outcome | Consisting of coronary heart disease, heart failure, stroke, and cardiovascular death | - |
| **Covariates** | | |
| Age | - | age_at_study_date_x100 |
| Sex | - | is_female |
| Marital status | Self-reported, Married / Widowed / Separated or divorced / Never married | marital_status |
| Education level | Self-reported, No formal school / Primary School / Middle School / High School / College / university | highest_education |
| Annual household income | Self-reported, <5,000 yuan / 5,000-9,999 yuan / 10,000-19,999 yuan / 20,000-34,999 yuan / ≥35,000 yuan | household_income |
| Smoking status | Self-reported, Never / Occasional / Past / Current | smoking_category |
| Drinking status | Self-reported, Never / Past / Current | alcohol_category |
| Physical activity (metabolic equivalent) | Self-reported | met |
| Diet | Self-reported  A healthy diet is defined as non-daily eating of vegetables, fruits, and eggs, and eating red meat daily or less than weekly. | Fresh.vegetables.frequenc  Perserved.vegetable.frequency  Fresh.fruit.frequency  Egg.frequency  Meat.frequency |
| Sleep duration | Self-reported, <= 6h / 7-8 h / >=9 h | sleep_hours |
| Hypertension | Self-reported medical history  SBP >= 140 mm Hg  DBP >= 90 mm Hg | Self-reported: hypertension_diag  SBP: sbp_mean  DBP: dbp_mean |
| Diabetes | Self-reported medical history  Fasting glucose >= 7.0 mmol/L  Random glucose >= 11.1 mmol/L | Self-reported: has_diabetes  Fasting glucose: fasting_glucose_x10  Random glucose: random_glucose_x10 |

BRI, body roundness index; DBP, diastolic blood pressure; ICD-10, International Classification of Diseases, Tenth Version; SBP, systolic blood pressure.

**Supplemental Table 2.** Baseline laboratory test results of study participants stratified by BRI quartiles (*n* = 9,336).

| **Characteristic** | **Overall**  *n* = 9,336 | **Q1 <2.66**  *n* = 2,239 | **Q2 2.66~3.39**  *n* = 2,179 | **Q3 3.39~4.22**  *n* = 2,324 | **Q4 >4.22**  *n* = 2,594 | ***P*-value** |
| --- | --- | --- | --- | --- | --- | --- |
| **Albumin**, g/L | 42.51 (2.77) | 42.22 (3.01) | 42.43 (2.78) | 42.66 (2.68) | 42.69 (2.60) | **<0.001** |
| **Creatinine**, μmol/L | 61 (53, 72) | 62 (54, 71) | 61 (52, 71) | 61 (52, 72) | 61 (52, 73) | 0.134 |
| **Uric acid**, μmol/L | 264 (217, 322) | 251 (207, 302) | 257 (212, 312) | 269 (218, 328) | 279 (228, 342) | **<0.001** |
| **ApoA1**, mg/dL | 129.67 (20.06) | 134.31 (21.34) | 131.36 (20.49) | 127.60 (19.17) | 126.10 (18.32) | **<0.001** |
| **ApoB**, mg/dL | 84.05 (21.04) | 75.12 (18.92) | 80.73 (19.76) | 86.96 (20.55) | 91.95 (20.75) | **<0.001** |
| **Total cholesterol**, mmol/L | 4.64 (0.98) | 4.35 (0.92) | 4.51 (0.90) | 4.74 (1.00) | 4.93 (1.01) | **<0.001** |
| **Triglycerides**, mmol/L | 1.58 (1.09, 2.41) | 1.15 (0.84, 1.58) | 1.44 (1.01, 2.11) | 1.78 (1.24, 2.63) | 2.17 (1.47, 3.23) | **<0.001** |
| **HDL-C**, mmol/L | 1.23 (0.30) | 1.34 (0.32) | 1.26 (0.30) | 1.19 (0.27) | 1.14 (0.25) | **<0.001** |
| **LDL-C**, mmol/L | 2.33 (0.70) | 2.11 (0.67) | 2.24 (0.65) | 2.40 (0.70) | 2.52 (0.71) | **<0.001** |
| **Lp(a)**, nmol/L | 17.77 (8.55, 44.21) | 19.43 (9.35, 45.00) | 17.77 (8.76, 44.43) | 17.19 (8.38, 46.50) | 16.67 (8.03, 40.40) | **0.001** |
| **hsCRP**, mg/L | 0.90 (0.41, 2.04) | 0.51 (0.25, 1.35) | 0.68 (0.34, 1.52) | 0.96 (0.48, 2.00) | 1.47 (0.78, 2.88) | **<0.001** |

Results are mean (standard deviation, SD) or median (interquartile range, IQR). ApoA1, apolipoprotein A1, ApoB, apolipoprotein B; HDL-C, high-density lipoprotein-cholesterol; hsCRP, hypersensitivity C-reactive protein; LDL-C, low-density lipoprotein-cholesterol; Lp(a), lipoprotein (a).

**Supplemental Table 3.** Relationship of obesity-related indices and cardiovascular outcomes.

| Obesity-related indices | Model 1  HR (95%CI) *P*-value | | Model 2  HR (95%CI) *P*-value | |
| --- | --- | --- | --- | --- |
| **Composite outcomes** | | | | |
| BRI | 1.34 (1.33-1.35) | <0.001 | 1.12 (1.12-1.13) | <0.001 |
| BMI | 1.15 (1.14-1.16) | <0.001 | 1.10 (1.09-1.11) | <0.001 |
| WHR | 1.20 (1.19-1.20) | <0.001 | 1.07 (1.06-1.08) | <0.001 |
| WC | 1.32 (1.31-1.33) | <0.001 | 1.14 (1.13-1.15) | <0.001 |
| HC | 1.18 (1.17-1.19) | <0.001 | 1.15 (1.14-1.16) | <0.001 |
| **CHD** | | | | |
| BRI | 1.40 (1.39-1.42) | <0.001 | 1.18 (1.16-1.19) | <0.001 |
| BMI | 1.24 (1.23-1.25) | <0.001 | 1.17 (1.16-1.18) | <0.001 |
| WHR | 1.19 (1.18-1.20) | <0.001 | 1.09 (1.08-1.10) | <0.001 |
| WC | 1.40 (1.39-1.42) | <0.001 | 1.22 (1.21-1.23) | <0.001 |
| HC | 1.33 (1.32-1.34) | <0.001 | 1.24 (1.23-1.26) | <0.001 |
| **HF** | | | | |
| BRI | 1.31 (1.28-1.35) | <0.001 | 1.12 (1.09-1.16) | <0.001 |
| BMI | 0.93 (0.90-0.96) | <0.001 | 1.03 (1.00-1.07) | 0.059 |
| WHR | 1.21 (1.19-1.23) | <0.001 | 1.09 (1.06-1.12) | <0.001 |
| WC | 1.13 (1.09-1.16) | <0.001 | 1.07 (1.04-1.11) | <0.001 |
| HC | 0.86 (0.83-0.89) | <0.001 | 1.01 (0.98-1.05) | 0.528 |
| **Stroke** | | | | |
| BRI | 1.35 (1.33-1.36) | <0.001 | 1.12 (1.11-1.13) | <0.001 |
| BMI | 1.17 (1.16-1.18) | <0.001 | 1.10 (1.09-1.11) | <0.001 |
| WHR | 1.20 (1.20-1.21) | <0.001 | 1.08 (1.07-1.09) | <0.001 |
| WC | 1.33 (1.32-1.35) | <0.001 | 1.13 (1.12-1.14) | <0.001 |
| HC | 1.18 (1.17-1.19) | <0.001 | 1.13 (1.12-1.14) | <0.001 |
| **Cardiovascular death** | | | | |
| BRI | 1.16 (1.15-1.18) | <0.001 | 1.01 (1.00-1.03) | 0.165 |
| BMI | 0.78 (0.77-0.79) | <0.001 | 0.87 (0.86-0.88) | <0.001 |
| WHR | 1.21 (1.20-1.21) | <0.001 | 1.02 (1.01-1.04) | 0.004 |
| WC | 1.05 (1.04-1.07) | <0.001 | 0.97 (0.95-0.98) | <0.001 |
| HC | 0.78 (0.76-0.79) | <0.001 | 0.92 (0.91-0.93) | <0.001 |

Model 1 is an unadjusted model. Model 2 is adjusted for age, sex, study region (urban/rural), marital status, educational level, household income, smoking, drinking, physical activity (metabolic equivalent), healthy diet, sleep duration, hypertension, and diabetes. BRI, body roundness index; BMI, body mass index; WHR, waist to height ratio; WC, waist circumference; HC, hip circumference; CHD, coronary heart disease; HF, heart failure; HR, hazard ratio; CI, confidence interval.

**Supplemental Table 4.** C-index (95%CI) for Cox models of obesity-related indices and cardiovascular outcomes.

| obesity-related indices | Composite outcome | CHD | HF | Stroke | Cardiovascular death |
| --- | --- | --- | --- | --- | --- |
| BRI | 0.588 (0.586-0.591) | 0.606 (0.602-0.609) | 0.566 (0.556-0.577) | 0.592 (0.590-0.595) | 0.529 (0.524-0.534) |
| BMI | 0.540 (0.538-0.543) | 0.564 (0.561-0.567) | 0.525 (0.514-0.535) | 0.547 (0.544-0.550) | 0.570 (0.566-0.575) |
| WHR | 0.576 (0.574-0.578) | 0.571 (0.568-0.574) | 0.585 (0.575-0.595) | 0.583 (0.580-0.586) | 0.580 (0.576-0.585) |
| WC | 0.580 (0.578-0.582) | 0.600 (0.597-0.603) | 0.528 (0.518-0.539) | 0.585 (0.582-0.588) | 0.508 (0.503-0.513) |
| HC | 0.548 (0.545-0.550) | 0.584 (0.581-0.587) | 0.548 (0.537-0.558) | 0.548 (0.545-0.551) | 0.573 (0.568-0.578) |

The Cox model adjusted for nothing. BRI, body roundness index; BMI, body mass index; WHR, waist to height ratio; WC, waist circumference; HC, hip circumference; CHD, coronary heart disease; HF, heart failure; CI, confidence interval.

**Supplemental Table 5.** Results of the sensitivity analyses.

| **Outcomes** | **As categorical variable,**  **Per one SD increase** | **As quartiles, vs Q1** | | |
| --- | --- | --- | --- | --- |
|  |  | **Q2 vs Q1** | **Q3 vs Q1** | **Q4 vs Q1** |
| **Excluding participants with outcomes within two years since follow-up (*n* = 9,087)** |  |  |  |  |
| Composite outcome | **1.12 (1.12-1.13)** | **1.07 (1.04-1.09)** | **1.18 (1.15-1.20)** | **1.37 (1.34-1.40)** |
| Coronary heart disease | **1.18 (1.16-1.19)** | **1.06 (1.03-1.10)** | **1.22 (1.18-1.26)** | **1.52 (1.47-1.57)** |
| Heart failure | **1.12 (1.09-1.16)** | 0.92 (0.83-1.02) | 1.00 (0.91-1.11) | **1.24 (1.13-1.37)** |
| Stroke | **1.12 (1.11-1.13)** | **1.17 (1.13-1.20)** | **1.28 (1.24-1.32)** | **1.41 (1.37-1.46)** |
| Cardiovascular death | 1.01 (1.00-1.03) | **0.93 (0.89-0.97)** | **0.86 (0.82-0.90)** | 0.96 (0.92-1.00) |
| **Fine and Gray’s competing risk model (non-cardiovascular death as the competing risk)** |  |  |  |  |
| Composite outcome | **1.12 (1.11-1.13)** | **1.07 (1.04-1.09)** | **1.18 (1.15-1.20)** | **1.37 (1.34-1.39)** |
| Coronary heart disease | **1.18 (1.16-1.19)** | **1.06 (1.03-1.10)** | **1.22 (1.18-1.26)** | **1.52 (1.47-1.56)** |
| Heart failure | **1.12 (1.08-1.16)** | 0.92 (0.83-1.01) | 1.00 (0.90-1.12) | **1.24 (1.13-1.37)** |
| Stroke | **1.12 (1.11-1.13)** | **1.17 (1.13-1.20)** | **1.28 (1.24-1.32)** | **1.41 (1.37-1.46)** |
| Cardiovascular death | 1.01 (0.99-1.03) | **0.93 (0.90-0.97)** | **0.86 (0.82-0.90)** | 0.96 (0.92-1.01) |
| **Additionally adjusting the use of antihypertensive drugs, lipid-lowering drugs and antidiabetic drugs (*n* = 11,191)** |  |  |  |  |
| Composite outcome | **1.12 (1.08-1.15)** | 1.06 (0.92-1.22) | **1.16 (1.02-1.32)** | **1.36 (1.19-1.54)** |
| Coronary heart disease | **1.17 (1.12-1.22)** | 1.06 (0.86-1.31) | **1.25 (1.03-1.52)** | **1.59 (1.31-1.92)** |
| Heart failure | **1.16 (1.03-1.31)** | 1.37 (0.72-2.60) | 1.26 (0.69-2.32) | 1.74 (0.97-3.12) |
| Stroke | **1.09 (1.05-1.13)** | 1.18 (0.98-1.42) | **1.25 (1.05-1.48)** | **1.34 (1.14-1.59)** |
| Cardiovascular death | **1.09 (1.05-1.13)** | 1.18 (0.98-1.42) | **1.25 (1.05-1.48)** | **1.34 (1.14-1.59)** |

**Supplemental Table 6.** Cox Model Analysis Adjusted for LDL-C (*n* = 9336).

| **Outcomes** | **BRI, as quartiles** | | | | **Continuous variable** | |
| --- | --- | --- | --- | --- | --- | --- |
|  | **Q2 vs Q1** | **Q3 vs Q1** | **Q4 vs Q1** |  | | **BRI, per SD increase** |
| Composite outcome | 1.06 (0.98-1.15) | **1.11 (1.02-1.20)** | **1.31 (1.21-1.42)** |  | | **1.11 (1.08-1.14)** |
| CVD | 1.12 (0.98-1.29) | **1.23 (1.07-1.41)** | **1.48 (1.30-1.70)** |  | | **1.16 (1.11-1.21)** |
| HF | 0.60 (0.32-1.14) | 0.91 (0.51-1.61) | 1.22 (0.71-2.11) |  | | 1.14 (0.95-1.37) |
| Stroke | 1.05 (0.95-1.15) | 1.08 (0.99-1.18) | **1.23 (1.12-1.34)** |  | | **1.09 (1.06-1.12)** |
| Cardiovascular death | 1.03 (0.91-1.17) | 1.05 (0.93-1.19) | 1.08 (0.95-1.22) |  | | **1.05 (1.01-1.10)** |

Results are HR (95% CI).


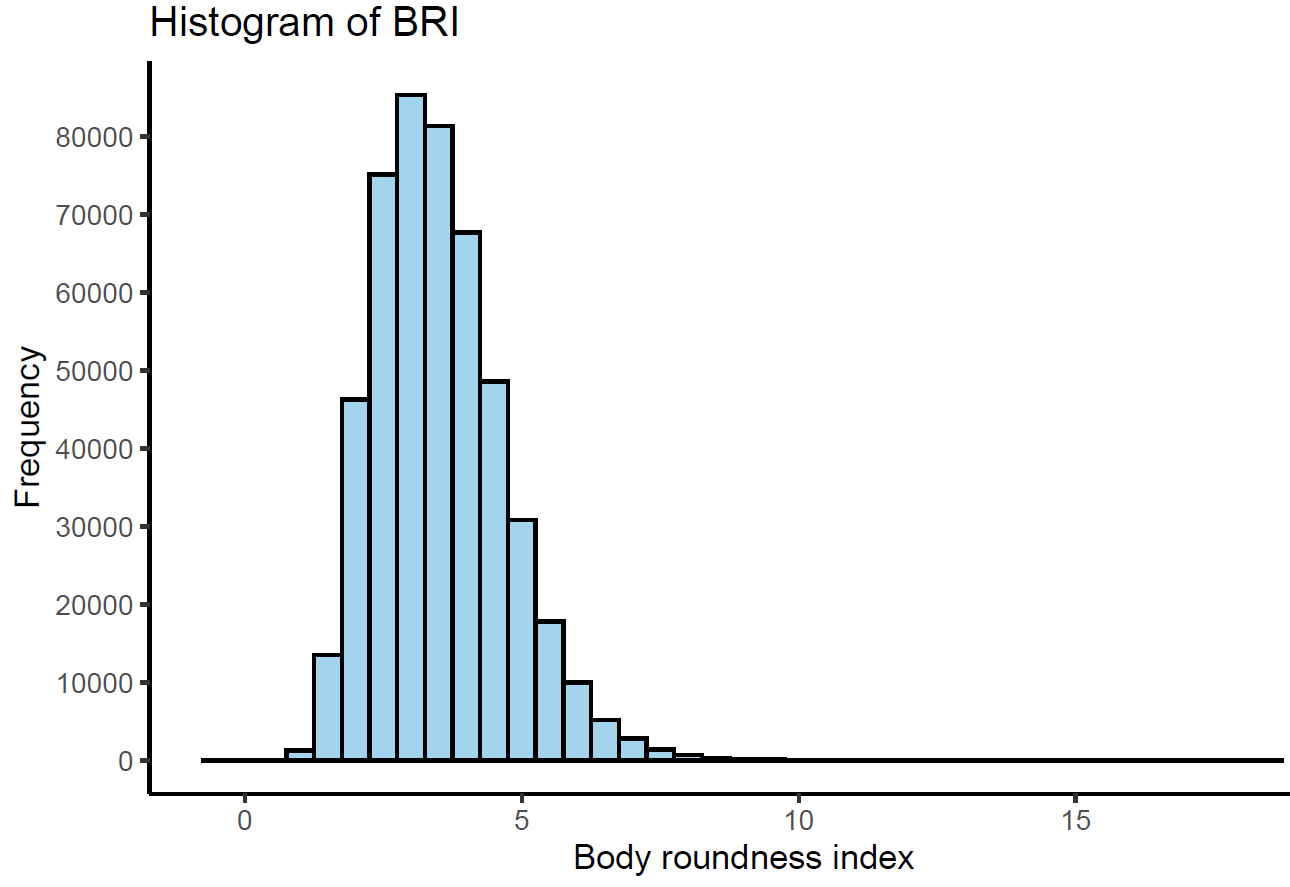


**Supplemental Figure 1.** Histogram of BRI in the CKB cohort.

BRI, body roundness index.


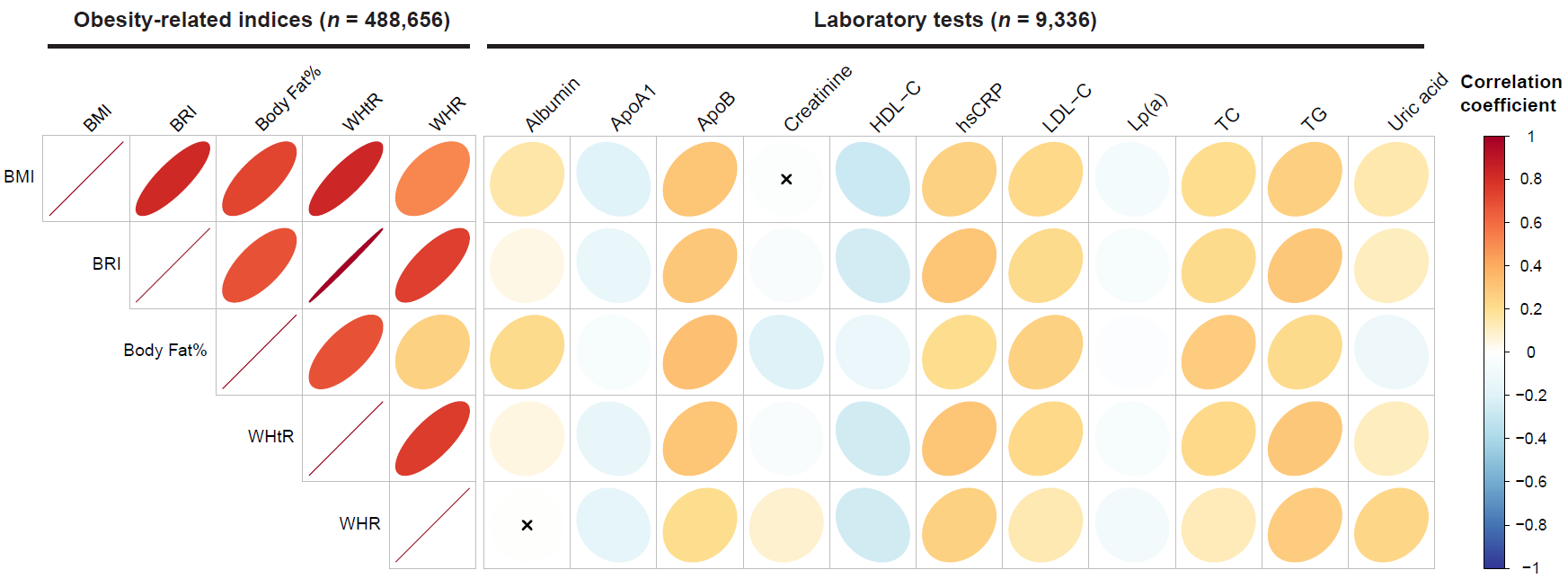


**Supplemental Figure 2.** Results of the correlation analysis.

ApoA1, apolipoprotein A1; ApoB, apolipoprotein B; BMI, body mass index; BRI, body roundness index; HDL-C, high-density lipoprotein-cholesterol; hsCRP, hypersensitivity C-reactive protein; LDL-C, low-density lipoprotein-cholesterol; Lp(a), lipoprotein (a); WHR, waist-hip ratio; WHtR, waist-height ratio.


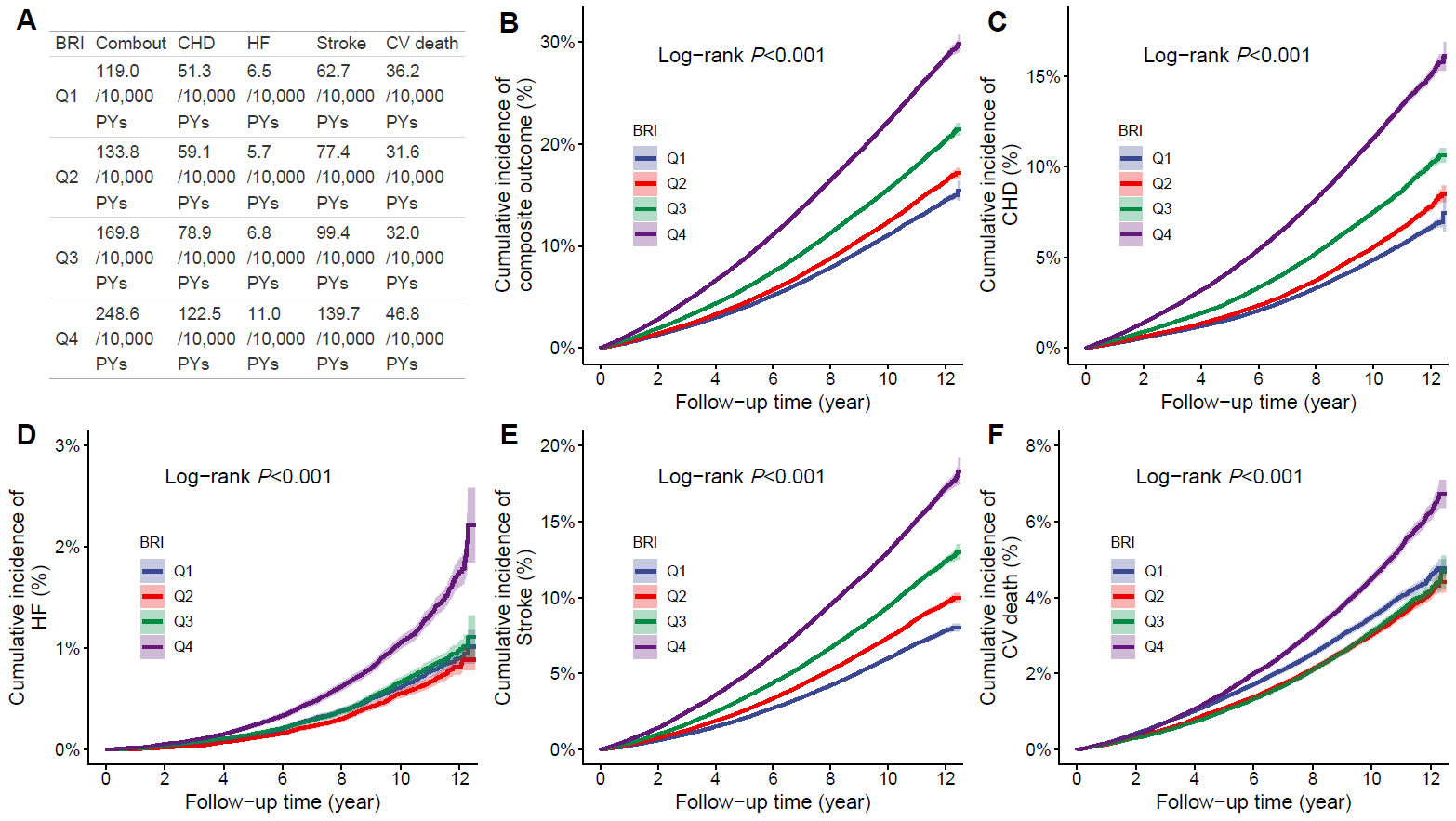


**Supplemental Figure 3.** Kaplan-Meier curves of BRI quartiles for cardiovascular outcomes.

(A) Incidence rate of study outcomes for each BRI quartile; (B-F) Kaplan-Meier curves of BRI quartiles for composite outcome (B), CHD (C), HF (D), stroke (E), and cardiovascular death (F). BRI, body roundness index; CHD, coronary heart disease; HF, heart failure.


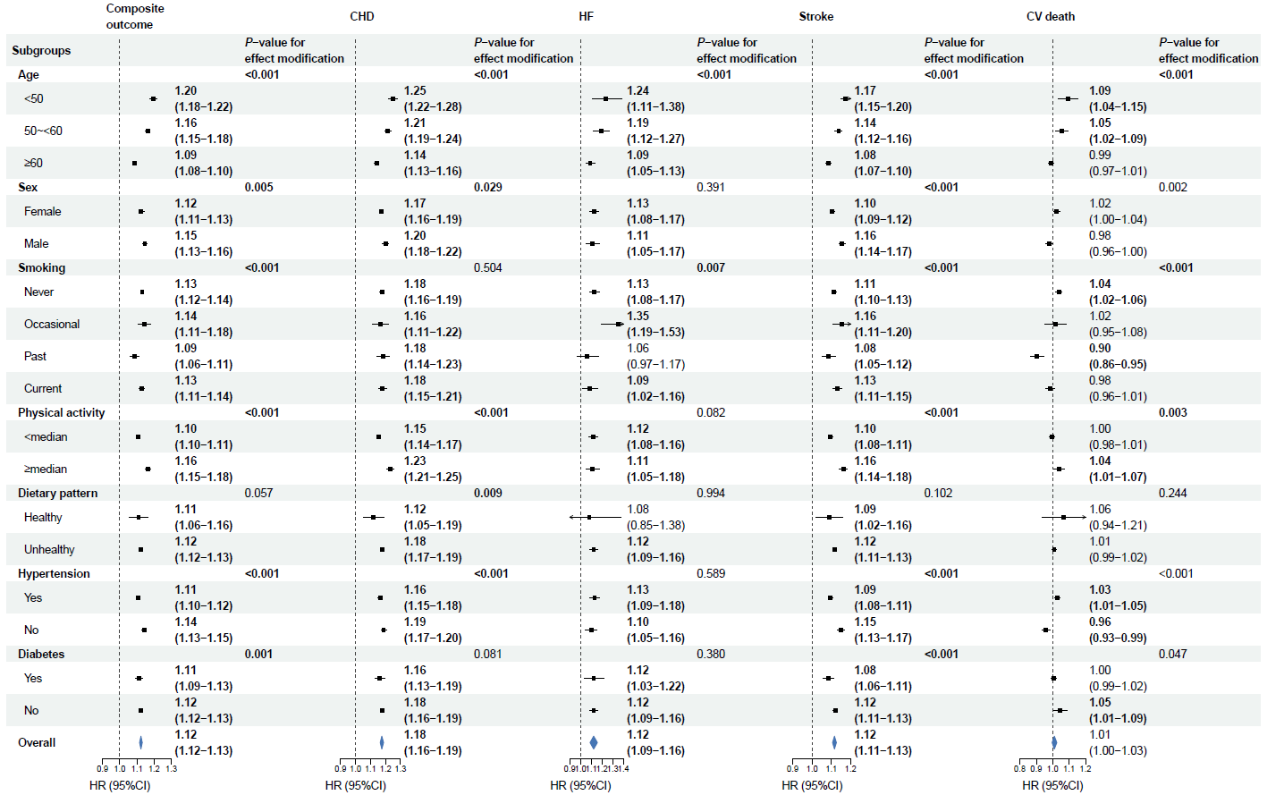


**Supplemental Figure 4.** Results of the stratified analysis.

The HRs (95%CIs) are adjusted for the same covariates as in Figure 2. CHD, coronary heart disease; CI, confidence interval; CV death, cardiovascular death; HF, heart failure; HR, hazard ratio.
